# Evidence-Based Complementary and Alternative Medicine in Current Medical Practice

**DOI:** 10.7759/cureus.52041

**Published:** 2024-01-10

**Authors:** Eman M Mortada

**Affiliations:** 1 Health Sciences Department, College of Health and Rehabilitation Sciences, Princess Nourah bint Abdulrahman University, Riyadh, SAU

**Keywords:** current practices, integrative medicine, evidence-based medicine, complementary medicine, alternative medicine

## Abstract

Despite the marvelous advancements in modern biotechnology and medical practices, the use of complementary and alternative medicine (CAM) is rapidly evolving and growing in the healthcare industry and has significantly increased in all modern societies. The health-seeking behavior of people, especially in developing countries, calls for bringing all CAM healers into the mainstream by providing them with proper training, facilities, and backup for a referral. Evidence-based CAM (EBCAM) therapies have shown remarkable success in treating diseases. It necessitates the integration of modern CAM systems in terms of evidence-based information sharing. Although a synergistic effect of interaction between the two systems works, large gaps in EBCAM still exist and are worth further studies to develop evidence for best CAM practices for the common goal of improving the health of people.

## Introduction and background

Over the past two decades, the use of complementary and alternative medicine (CAM) has dramatically expanded in all modern societies and is expanding and growing more quickly than ever before in the healthcare sector [1]. Since there is no single definition of CAM that is accepted by all, multiple definitions of CAM are reported in different pieces of literature. It is "diagnosis, treatment, and/or prevention that complements conventional medicine by adding to a common good, addressing a need not addressed by conventional wisdom, or broadening the conceptual frameworks of medicine" [2]. Complementary and alternative medicine is defined according to the National Center for Complementary and Integrative Health (NCCIH) as “a group of diverse medical and health care systems, practices, and products that are not presently considered to be part of conventional medicine” that uses the usual methods of healing or treating disease that is taught in Western medical schools [3-6]. Conventional medicine is defined by the National Cancer Institute (NCI) of the National Institutes of Health (NIH) as “a system in which health professionals who hold an MD or DO degree treat symptoms and diseases using drugs, radiation, or surgery” [4].

Complementary and alternative medicine is expanding fast in the healthcare sector and has grown significantly in all modern societies over the past 20 years, despite all the wonderful advances in modern biotechnology and medical practices that have never been seen before. People's tendency to seek health care, particularly in developing nations, necessitates integrating all CAM practitioners into mainstream medicine by giving them access to facilities, adequate training, and support systems in case of urgent referrals. Treatment outcomes for diseases with evidence-based complementary and alternative medicine (EBCAM) have been remarkably successful. To share evidence-based information, contemporary CAM systems must be integrated. Today, many patients seek healing through both alternative and conventional medicine. So the objective of the current literature review is to shed light on the use of CAM while highlighting its current status in Saudi Arabia.

## Review

Alternative medicine

In certain nations, the terms 'traditional medicine' and 'complementary medicine' are interchangeable. According to the NCCIH, alternative health is defined as when “a non-mainstream approach is used in place of conventional medicine.” The NCCIH defines complementary health as “a non-mainstream approach used together with conventional medicine.” Therapies that are utilized in conjunction with conventional medicine and traditional Western medicine are referred to as complementary therapies. Therapeutic methods employed in place of traditional medicine are referred to as alternative medicine [5]. Traditional medicine is described by the WHO as "various health practices, approaches, knowledge, and beliefs, including plant, animal, and/or mineral-based medicines, spiritual therapies, manual techniques, and exercises employed alone or in combination to preserve well-being, in addition to treating, diagnosing, or preventing illness" [7].

All therapeutic modalities that are not part of standard medical practice are included in alternative medicine (AM). It consists of more than 160 techniques or remedies [8]. It can be categorized into pharmacological categories (such as herbalism or homeopathy), physical therapies (such as acupuncture, cupping, or chiropractic), nutritional approaches (such as macrobiotics and vegetarianism), or cognitive therapy (e.g., hypnosis and other methods). Complementary and alternative medicine is often used as an umbrella term to describe a wide range of both common and more obscure modalities. They range from "medical interventions that are not taught in medical schools or generally available in hospitals” to "strategies that have not met the standards of clinical effectiveness, either through randomized controlled clinical trials (RCTs) or through the consensus of the biomedical community" [8-10].

Integrative medicine

Integrative medicine (IM) is a diverse range of conventional and complementary therapies that refers to simply incorporating CAM into biomedicine in conventional care. It aims to combine conventional medicine with alternative therapies to create a more efficient and cost-effective healthcare delivery system. Conventional medicine relies on methods proven to be safe and effective through carefully designed trials and research. Integrative medicine is characterized as being patient-centered, taking into consideration the whole person, i.e., body, mind, and spirit. It places a focus on the therapeutic alliance and employs all necessary treatments, including the traditional and alternative.

The term CAM was replaced by the term 'integrative medicine' in the 1990s. Methods that had not previously been recognized as legitimate treatments in Western medicine are now referred to as IM. According to 2002 research by the National Center for Health Statistics (NCHS) of the CDC, 62% of individuals utilized some type of IM therapy for certain health benefits in the previous year [11]. Natural products or herbal drugs rank among the top 10 most popular remedies in IM that people in the US use most regularly, as many people believe that herbal therapies are natural, safer, and healthier than allopathic medications [12]. Table 1 shows the most widely used IM therapies in the US.

**Table 1:** The top 10 most popular IM therapies used in the US The data in this table is sourced from the studies of Barnes et al. and Devine et al. [11,12]. IM: Integrative medicine

Ranking	Most commonly used IM remedies	Licensure status	Systemic review(s) of evidence
1	Natural products/herbal medications	Supplements are exempt from FDA approval	Available
2	Deep breathing	Can be advised by any type of provider or self-taught	Available
3	Meditation	Can be advised by any type of provider or self-taught	Available
4	Osteopathic manipulation and chiropractic treatment	Licensure needed for DO and DC in the USA	Available
5	Massage	Licensure is required in most of the US	Available
6	Yoga	Certification is available, no formal licensure is needed in the USA	Available
7	Diet-based therapies	None unless consulting as a certified dietician or licensed naturopath	Depends on the diet studied
8	Progressive relaxation	No formal licensure/certification required	Available
9	Guided imagery	No formal licensure/certification required	Available
10	Homeopathic therapy	Certification/diploma but no licensure required	Available

The growing availability and acceptance of IM by the general public, concerns about the use of allopathic medications, a rise in the demand for preventative medicine, and patient self-advocacy for alternatives to allopathic medications that fail to treat conditions or cause side effects contribute to the popularity of herbal medications [12]. Table 2 lists additional IM therapy modalities that physicians should be familiar with.

**Table 2:** Additional IM therapy modalities that physicians should be familiar with The data in this table is sourced from the studies of Barnes et al. and Devine et al. [11,12]. IM: Integrative medicine

IM modalities	Licensure status	Systemic review(s) of evidence
Acupuncture	Required	Available
Aromatherapy	Not required	Limited
Ayurveda	Not required	Available
Biofeedback	Not required	Available
Energy psychology	Not required	Available
Environmental medicine	Not required	Available
Feldenkrais	Not required	Limited
Hypnotherapy	Required	Available
Naturopathy	Required	Available

Herbal medicine can be challenging due to the inherent characteristics of plants, which include an extensive diversity of chemical constituents and the potential for different effects from plant parts. Also, it is frequently unknown which of the components is in charge of producing the intended effect. Some of the compounds that might result in herb-drug interactions are well known. In addition to side effects and contraindications for every single herb, it's crucial to ask patients directly about all medications and therapies they've taken, whether they were prescribed or not, especially since people may not disclose to their doctors that they take herbal remedies out of concern that this practice will not be allowed. Additionally, as CAM is growing and people are seeking out and using these methods, family doctors might benefit from knowledge of this area.

Table 3 displays the indications, contraindications, and side effects of the most frequently used herbal remedies [13]. Complementary and alternative medicine treats the patient as a whole, i.e., treating the mind, body, and spirit holistically to balance them based on the body's interconnection, rather than concentrating on specific diseases or body systems. We must always keep in mind that health is a complete condition of physical, mental, and social well-being and not only the absence of disease or disability. Without putting the needs of the whole person first and utilizing all appropriate and research-based therapy and lifestyle modalities, medical practitioners, and disciplines, it is impossible to accomplish this definition of health [13].

**Table 3:** Indications, contraindications, and side effects of commonly used herbal remedies The data for this table is sourced from the study by Bent and Ko [13]. TB: Tuberculosis, MS: Multiple sclerosis, MAOI: Monoamine oxidase inhibitors, GI: Gastrointestinal, PO: Per os

Common herbs	Indications	Contraindications	Side effects	Systemic review(s) of evidence
Echinacea	Promotes natural resistance to infection; is used mainly in upper respiratory infections	Systemic illnesses such as HIV, TB, and MS	Chills, fever, nausea, allergic reaction	Yes
Garlic	Hyperlipidemia	None	Avoid before surgery; potentiates warfarin	Yes
Ginkgo biloba	Dementia, cognitive impairment	A known hypersensitivity to ginkgo; caution in depression	Stomach upset, headache, skin reaction	Yes
Saw palmetto	Symptoms of benign prostatic hypertrophy	None known	Gastric upset	Yes
Ginseng	Fatigue, weakness, physical performance	Hypertension, excessive caffeine use	Possible interaction with MAOI	Yes
Grape seed extract	Venous insufficiency, possibly with certain types of cancers	A known allergy to grapes	Headache, itchy scalp, dizziness, and/or nausea	No
Green tea	Cancer treatment, cognitive impairment, GI illnesses	Can interact with many medications	In excess can cause the same side effects as caffeine	No
St. John’s Wort	Mild to moderate depressive mood, anxiety	Not recommended with other antidepressants	Trouble sleeping, vivid dreams, fatigue	Yes
Bilberry	Vision impairment	Caution with diabetic and anticoagulant medications	Rash	No
Aloe	Dermatitis, wound healing	As PO supplement can act as a laxative	Burning/skin irritation	Yes

Complementary and alternative medicine is predicated on the idea that a person's body is capable of healing itself. It places a greater emphasis on health restoration than disease therapy and sees disease as a symptom of a change in the way the body naturally heals itself [14]. The commonly practiced traditional and complementary medicine in the Middle East is folk medicine, comprising self-medication and simple herbal remedies, as well as traditional therapies such as unani, ayurveda, bone setting, and massage [15].

Conflict between CAM and conventional medicine

Conventional medicine and CAM have long been at odds. Since many conventional physicians often refer their patients to CAM practitioners, the relationship is a little more flexible today [16]. Complementary and alternative medicine is currently widely used and frequently used in conjunction with conventional medical treatments. Integrative medicine refers to the practice of patients combining conventional medical care with CAM, proving that the two medical philosophies do not necessarily have to be at odds with one another. Nowadays, the national health systems of Egypt, Jordan, Kuwait, Saudi Arabia, and the UAE integrate herbal CAM alongside Western treatment [17]. Its widespread use has affected health professionals, researchers, and policymakers, in addition to its users.

Complementary and alternative medicine healers must be integrated into society by providing them with facilities, training, and referral support, given people's propensity to seek them out, especially in developing countries. Developed nations have policies and advocacy groups to help them better integrate CAM practices into their systems of national health care delivery [18]. Similarly, certain nations, such as China, South Korea, India, Singapore, and Hong Kong, have developed IM that uses both CAM and conventional treatments that are safe and successful [19-21]. Complementary and alternative medicine is commonly used by adults for the management of chronic conditions such as chronic pain alleviation, musculoskeletal and respiratory diseases, diabetes, psychiatric problems, and life-threatening conditions such as cardiovascular diseases and cancer that are costly to society [22]. Traditional Chinese medicine (TCM) uses a wide range of these healing modalities, many of which lack factual support, to attempt to avoid or treat disease.

The increasingly wide use of CAM

Over the past two decades, CAM has become widely used in all modern societies and communities for a wide range of clinical disorders because of its growing popularity and cultural acceptance. The justifications for CAM usage by the general public vary between nations, particularly individuals who reside in rural areas. The reasons for including CAM practitioners in healthcare facilities are multifaceted: first, economic factors; second, the power of traditional beliefs; third, the fact that the practitioners are well-respected and knowledgeable in their field; and, fourth, the dearth of health experts [1]. The acceptance of CAM use, either as a supplementary treatment or an alternative, in patients with diseases depends on a variety of factors, including the condition or disease, illness, and medications. Some patients seek CAM therapies because they are dissatisfied with conventional medicine or because they have heard from others that CAM is beneficial in treating specific disorders. Some patients do not trust mainstream medicine because they believe CAM has fewer adverse effects [23]. Dissatisfaction with severe adverse effects and the high costs of allopathic medication lead patients to use CAM. They are motivated by the cooperation, emotional support, empathetic attitude, humanistic care, and active listening offered by CAM traditional healers compared to modern allopathic practitioners [24].

Most CAM patients find that CAM treatments correspond with their values and views about health and life; however, they still anticipate that their healthcare providers will support them in making treatment decisions based on formal evidence or clinical practice [25]. Patients seldom ever consult their doctors about CAM or even share information about their usage of CAM since they strongly believe that their clinicians have the power to forbid CAM use. Another element influencing the rising interest in CAM is a patient-centered strategy that equips patients with the knowledge to make better decisions [26].

The status of CAM in Saudi Arabia

As part of its 2030 strategy, Saudi Arabia is undergoing a healthcare reform [27]. Without a holistic healthcare strategy, the current healthcare system will not be able to handle the present and future burden of lifestyle illnesses. The key driving force behind the search for an alternative medical system to address the unmet needs in contemporary medicine is to meet the problems posed by the rising incidence of noncommunicable diseases and to preserve the caliber of healthcare services. Saudi Arabian researchers, medical professionals, decision-makers, and the general public have all shown a significant increase in interest in the use of traditional and complementary therapies in recent years. In evaluating the use of CAM in Saudi Arabia, the Ministry of Health notes that the free health care provided to its nationals does not include CAM services. In response to a ministerial decree (No. 236) dated 10/8/1429 H (12/8/2008 G), a center for CAM was established. The center is not only a reference center for all issues related to CAM but also regulates CAM practices within the healthcare system and supports the use of EBCAM as a complementary addition to conventional medicine [28]. The practices of CAM are usually related to the consumers' religious beliefs. Consequently, the common practices are therapy according to the Holy Quran, using honey and black seed in addition to prophetic medicine in the form of alhijama (cupping) [29]. Recently, modern practices in the private sector introduced acupuncture to the Saudi community through a well-established clinic [30]. In the Western world, in contrast to Saudi Arabia, the commonly used types of CAM are relaxation techniques, ginseng, chiropractic, osteopathy, massage, mineral supplements, and homeopathy [31].

The evidence-based medicine (EBM) movement

According to ongoing recommendations from the WHO, complementary medicine practitioners should be integrated into the healthcare system, when necessary, to increase the safety and efficacy of their patients' use of complementary therapies [32]. The EBM movement has gained ground in the medical community since the 1990s. Along with the rise in the use of CAM, there has been a boom in CAM-related research, literature, and evidence-based practices. Evidence-based medicine is described by Stacks as "the thoughtful, explicit, and prudent application of the best available evidence in making decisions concerning the care of specific patients" [33]. It emphasizes the use of data from carefully planned and executed studies to facilitate decision-making [34]. Also, it has become a crucial component of contemporary medical care. Evidence-based medicine improves health by empowering patients. Patients are given the freedom to select the preferred way of receiving health care while being informed about the safety and efficacy of their preferred technique [35]. The EBM movement aims to minimize biases that would otherwise result in subpar treatment and support the scientific basis of medicine.

To lessen the amount that clinical medicine depends on heuristics and haphazard, experience-based clinical competence, EBM studies are meant to provide a firmer evidence base for medical practice. Initially, EBM was developed as a countermeasure against the shortcomings and untrustworthiness of doctors' own experience. Clinical modalities that can be utilized as adjuncts to conventional medicine should be known to healthcare professionals (HCPs) to improve medical practice and be more responsive to patients' preferences and requirements [36]. The fundamental tenets of IM are to take into account all variables that affect health, wellness, and disease; partner with the patient and practitioner in the healing process; and use IM appropriately to support the body's natural healing processes [37].

Moreover, recommendations for choosing IM practitioners via referral sources should be examined. Family physicians would be wise to learn about this field, given how it is expanding and how patients are requesting and using these approaches. Traditional medical practices have been utilized successfully for a variety of disorders since antiquity, and they have persisted and thrived despite the disorganized state of research into holistic system methods. However, over the past few decades, research has started to empirically explore the efficacy of conventional treatment modalities in a variety of diseases and the underlying mechanisms of action. This research includes RCTs, systematic reviews, meta-analyses, and observational studies into CAM [38]. Many CAMs are rejected by traditional medicine since the efficacy of the treatment is not shown through a double-blinded RCT, and conventional pharmaceuticals are only let onto the market when their clinical trials demonstrate their efficacy [39]. Many of the questions regarding the safety and efficacy of these medicines will be resolved as research advances [40]. Contrary to popular belief, there is more complementary medical research than is commonly recognized due to a stronger appreciation of the research's importance among complementary practitioners. The Cochran collection includes more than 150 reviews of CAM and about 6,000 pieces of randomized research.

The EBCAM therapies have proven to be effective, highlighting the necessity of integrating CAM and mainstream systems in terms of the exchange of evidence-based knowledge. The two systems' interaction has a synergistic effect with the joint goal of improving human health [38]. Several research institutions and international health organizations encourage the use of research and training in traditional medicines throughout the world. Healthcare professionals must be aware of their patients' concurrent, widespread use of CAM and alter their attitudes toward it. Health education seminars promote CAM dialogue between HCPs and their patients by enhancing understanding and attitudes regarding CAM. A collection of public health policies is required to give decision-makers and program designers examples and starting points.

It is advisable to incorporate the CAM curriculum into the integrative medical curriculum to build up strong theoretical and empirical knowledge regarding the appropriate uses of CAM among medical students and prepare them to prescribe effectively and efficiently in the future. This can be guided by the experience of many medical universities in the Western world that have already incorporated the CAM curriculum as an elective or mandatory course [40]. In Saudi Arabia, where low back and neck pain, depressive disorders, migraine, diabetes, and anxiety disorders cause the most impairment, it is imperative to encourage healthy lives and promote well-being for all ages through the expansion of an integrated health strategy.

## Conclusions

Complementary and alternative medicine has gained a lot of traction in the medical sciences and is now seen as an essential part of the healthcare system. Complementary and alternative medicine is employed in developing countries, both within and outside the formal healthcare system. This emphasizes the need for its regulation, with a focus on training and research. National healthcare systems now incorporate CAM recommendations and a policy framework. To enable healthcare providers to counsel patients on the safe use of CAM, it is imperative that EBCAM practices be regulated and promoted, and that efforts be made to enhance the knowledge and perception of CAM among these experts. Thus, physicians need to have nonjudgmental conversations with their patients about CAM. Health education programs must be initiated to enhance participants' understanding of CAM and promote conversations on the same between healthcare providers and their patients. To give planners, legislators, and program creators models and points of reference, a collection of public health and policy viewpoints is necessary. It is advisable to include CAM therapies in the IM curriculum and to ensure that medical students have a solid foundation of information regarding the recommendations and applications of CAM. When CAM practices are combined with high-quality scientific data, patients are empowered to make better decisions for the delivery of optimal healthcare.
